# Transient Liquid Phase Bonding Using Sputtered Cu/Sn Multilayers

**DOI:** 10.3390/ma19214440

**Published:** 2026-10-22

**Authors:** Dong-Yurl Yu, Junhyuk Son, Yun-Chan Kim, Yong-Ho Ko, Tae-Young Lee, So-Jeong Lee, Dongjin Byun, Junghwan Bang

**Affiliations:** 1 Advanced Packaging Integration Center, Korea Institute of Industrial Technology, 156 Gaetbeol-ro, Yeonsu-gu, Incheon 21999, Republic of Korea; alpha0987@kitech.re.kr (D.-Y.Y.); jhson@kitech.re.kr (J.S.); kyc20@kitech.re.kr (Y.-C.K.); yonghoko@kitech.re.kr (Y.-H.K.); flthwha27@kitech.re.kr (S.-J.L.); 2 Department of Material Science and Engineering, Korea University, Anam-dong, Seongbuk-gu, Seoul 136-713, Republic of Korea; dbyun@korea.ac.kr; 3 Center for Cooperative Research Facilities, Tech University of Korea, 65 Emtibeuibukro-ro, Siheung-si 15119, Republic of Korea; lty1226@tukorea.ac.kr; 4 Department of Materials Science and Engineering, Inha University, 100 Inha-ro, Michuhol-gu, Incheon 22212, Republic of Korea

**Keywords:** transient liquid phase bonding, Cu/Sn multilayers, Cu_3_Sn, shear strength, high temperature reliability

## Abstract

Power electronic modules require bonding materials capable of maintaining high reliability during high-temperature operation. In this study, transient liquid phase (TLP) joints were fabricated using sputtered Cu/Sn multilayers, and their mechanical performance was evaluated through isothermal aging at 175 °C for up to 500 h and die-shear testing. The effects of the initial Cu/Sn multilayer configuration on interfacial microstructural evolution and shear strength were systematically investigated. In the as-bonded state, Cu_6_Sn_5_ and Cu_3_Sn intermetallic compounds (IMCs) were formed in the three primary configurations, while localized cracking was observed in an additional 7layer/7layer configuration examined only in the as-bonded state. During aging, the Cu_6_Sn_5_ to Cu_3_Sn transformation strongly depended on the initial multilayer configuration, although the three primary configurations were completely transformed into Cu_3_Sn after 500 h. A comparative kinetic analysis suggested a higher apparent Cu_3_Sn growth rate for the 5layer/5layer configuration, although this trend was derived from a limited number of aging time points. The 5layer/5layer joints exhibited the highest initial shear strength, which was attributed to the presence of an island-shaped Cu_3_Sn region within the bonding layer. After 500 h of aging, however, the 3layer joints exhibited the highest shear strength, consistent with their lower interfacial void fraction, whereas the 5layer/5layer joints exhibited the greatest void accumulation. These results demonstrate that the initial Cu/Sn multilayer architecture plays an important role in IMC transformation, interfacial void evolution, and shear strength evolution during isothermal aging at 175 °C. Therefore, controlling the Cu/Sn multilayer configuration provides a potential approach for tailoring the microstructure and mechanical performance of Cu3Sn joints under the investigated aging conditions.

## 1. Introduction

The demand for power electronic modules used in electric vehicles (EVs), hybrid electric vehicles (HEVs), and renewable energy systems has increased rapidly in recent years. In particular, wide-bandgap semiconductors, such as silicon carbide (SiC) and gallium nitride (GaN), have attracted considerable attention owing to their superior power conversion efficiency and ability to operate at temperatures exceeding 300 °C [1,2,3,4,5,6]. Under these severe operating conditions, the reliability of die-attach materials plays a critical role in determining the performance and lifetime of power electronic modules. Therefore, bonding materials with excellent thermal stability and mechanical reliability are essential for high-temperature power electronic packaging.

Conventionally, Pb-Sn solders have been widely employed for die attachment because of their excellent processability and wettability. However, environmental regulations have accelerated the replacement of Pb-containing solders with lead-free alternatives, including Sn-Ag, Sn-Ag-Cu, Sn-Zn, and Sn-Cu alloys [7,8]. Although these lead-free solders exhibit satisfactory mechanical properties, their relatively low melting temperatures limit their application in high-temperature power devices based on SiC and GaN. High-temperature solders, such as Au-Sn and Sn-Sb alloys, have been proposed as alternatives; however, the practical application of Au-Sn solder is limited by its high material cost, whereas the broader application of Sn-Sb solder can be constrained by its relatively brittle mechanical behavior and more limited processing flexibility compared with conventional Sn-based solders [9]. To overcome these limitations, several high-temperature bonding technologies, including Ag sintering, Cu sintering, and transient liquid phase (TLP) bonding, have been extensively investigated [10,11,12,13,14,15,16,17,18].

Among these technologies, Cu-Sn TLP bonding has attracted considerable attention because bonding can be performed at relatively low temperatures while producing intermetallic compound (IMC) joints with substantially higher remelting temperatures after bonding [19,20,21,22]. During TLP bonding, molten Sn reacts rapidly with Cu to form Cu_6_Sn_5_, followed by the formation and growth of Cu_3_Sn through continued interdiffusion. As Sn is progressively consumed, the initially Sn-containing bonding layer is converted into a high-melting-point IMC joint. In particular, Cu_3_Sn is attractive for high-temperature interconnection because of its high thermal stability after complete reaction. Therefore, controlling the formation and transformation of Cu-Sn IMCs is one of the key factors determining the processing efficiency and long-term reliability of Cu-Sn TLP joints.

Despite these advantages, conventional Cu-Sn TLP bonding still presents important processing and reliability challenges. Complete consumption of Sn and transformation of Cu_6_Sn_5_ into Cu_3_Sn may require prolonged bonding or subsequent thermal exposure because the reaction becomes increasingly controlled by solid-state diffusion as the IMC layers grow. Prolonged interdiffusion can also promote vacancy accumulation and Kirkendall void formation, particularly in association with Cu_3_Sn growth, thereby reducing the effective bonding area and degrading the mechanical integrity of the joint [23,24,25]. Consequently, an appropriate interlayer design should provide a short diffusion distance for rapid Cu_3_Sn formation while suppressing excessive vacancy accumulation and interfacial void formation during subsequent high-temperature exposure. Previous studies have reported that thermal conditions affect IMC growth behavior, microstructural evolution, and mechanical properties in Sn-based solder joints [26,27].

Cu/Sn multilayer structures provide a potential strategy for achieving this balance. Dividing the Cu and Sn interlayers into multiple thin layers increases the number of Cu/Sn interfaces and shortens the local diffusion distance, allowing the initial reaction pathway and IMC distribution to be tailored during TLP bonding. Sputter deposition is particularly suitable for this approach because thin Cu and Sn layers with controlled thicknesses and well-defined interfaces can be sequentially fabricated [28]. However, increasing the number of Cu/Sn interfaces may also alter the spatial distribution of Cu_6_Sn_5_ and Cu_3_Sn and the subsequent diffusion paths during thermal exposure. These differences can affect not only the rate of Cu_6_Sn_5_ to Cu_3_Sn transformation but also vacancy accumulation, void development, fracture paths, and ultimately the mechanical reliability of the joint. Therefore, the number and arrangement of Cu/Sn layers should be considered not merely as processing parameters for accelerating TLP reactions but as structural variables governing the long-term evolution of the bonding layer.

Previous studies on Cu-Sn TLP bonding have predominantly focused on accelerating IMC formation, reducing bonding time, or achieving complete Sn consumption. In comparison, the influence of the initial Cu/Sn multilayer configuration on the entire sequence of microstructural evolution remains insufficiently understood. In particular, the relationship between the transformation from the as-bonded Cu_6_Sn_5_/Cu_3_Sn structure to a fully developed Cu_3_Sn joint, interfacial void formation, and mechanical degradation during prolonged thermal aging has not been systematically clarified. A systematic comparison of Cu/Sn multilayer configurations is therefore necessary to clarify how differences in multilayer architecture, including the number of Cu/Sn interfaces and the resulting total interlayer thickness, affect the microstructural evolution and long-term reliability of TLP joints.

Accordingly, in this study, TLP joints were fabricated using sputtered Cu/Sn multilayers with different multilayer configurations. Three primary configurations, namely 3layer, 3layer/3layer, and 5layer/5layer, were systematically evaluated through isothermal aging at 175 °C for up to 500 h. The evolution of Cu_6_Sn_5_ and Cu_3_Sn was investigated together with the quantitative evolution of interfacial voids, and the corresponding changes in mechanical reliability were evaluated through die-shear testing and fracture-surface analysis. In addition, a 7layer/7layer configuration was examined in the as-bonded state to further explore the microstructural consequences of increasing the multilayer architecture beyond the primary configurations. By correlating the initial multilayer architecture with IMC transformation, interfacial void evolution, fracture behavior, and shear strength, this study aims to clarify the role of Cu/Sn multilayer configuration in determining the long-term reliability of TLP joints and to provide practical guidance for selecting suitable multilayer architectures for high-temperature power electronic packaging.

## 2. Experimental Procedure

Cu/Sn multilayers were deposited onto Si chips and direct-bonded copper (DBC) substrates by sputtering. The Si chips measured 3 mm × 3 mm, whereas the DBC substrates measured 17.8 mm × 12.7 mm. The DBC substrate consisted of a 300 μm thick Cu layer with an organic solderability preservative (OSP) surface finish. Prior to multilayer deposition, the Si chips were sequentially coated with a 50 nm thick Ti adhesion layer and a 5 μm thick electroplated Cu layer.

Prior to Cu/Sn multilayer deposition, Ar plasma pre-cleaning was performed for 15 min, followed by pre-sputtering for 10 min. The detailed sputtering conditions are summarized in Table 1. The base pressure of the sputtering chamber was maintained below 1 × 10^−5^ Torr, while the working pressure during deposition was maintained at 3.6 × 10^−3^ Torr under an Ar atmosphere. During Cu and Sn deposition, the direct current (DC) current and sputtering power were maintained at 0.3 A and 80 W, respectively. The deposition rates of Cu and Sn were approximately 25.0 and 41.7 nm/min, respectively.

**Figure 1:**
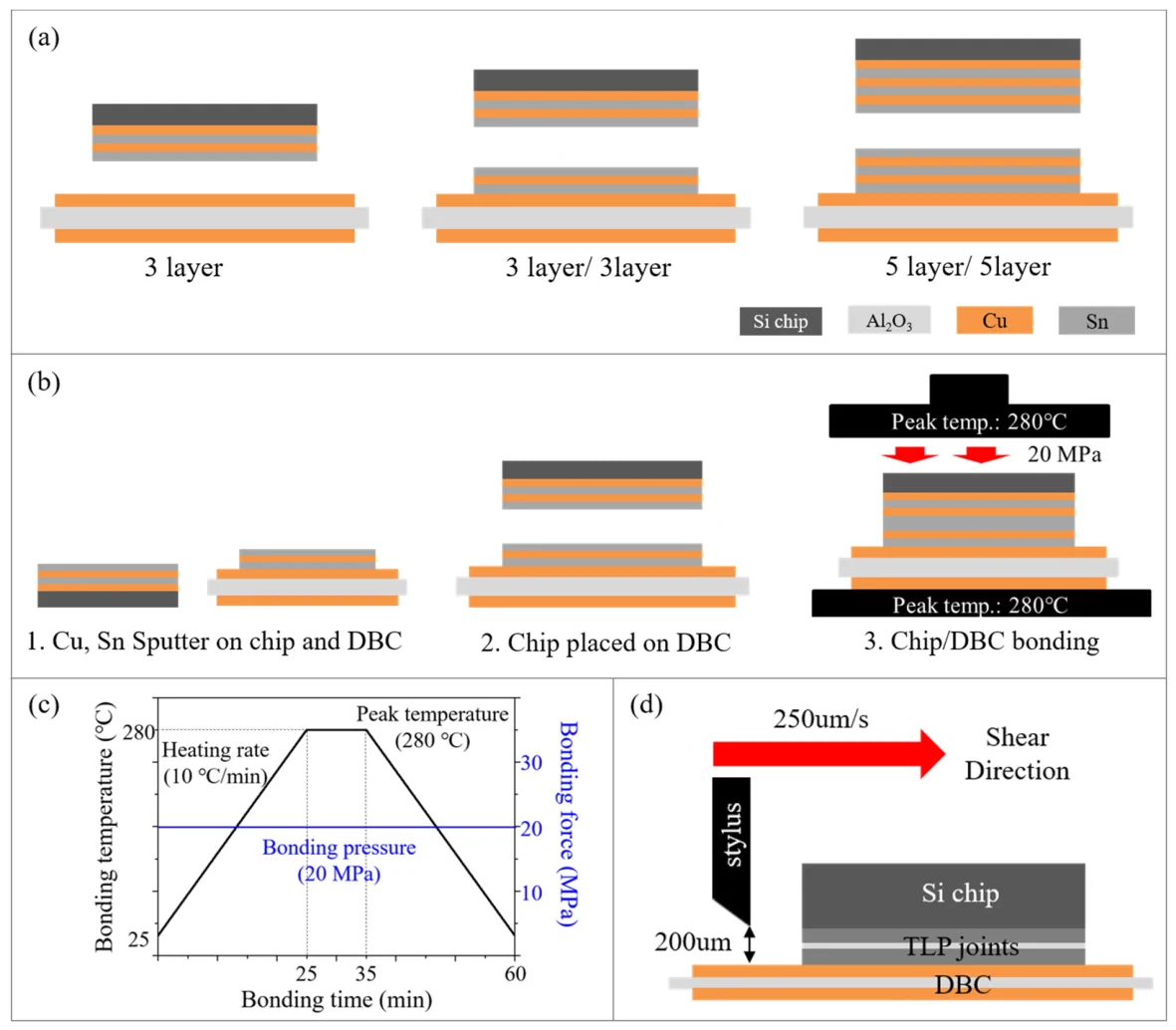
Schematic illustration of the experimental procedure: (**a**) preparation of the Cu/Sn multilayer on the Si chip and DBC substrate, (**b**) schematic of the TLP bonding process, (**c**) bonding temperature and pressure profile, and (**d**) shear test configuration for the TLP joints.

As illustrated in Figure 1a, three primary multilayer configurations, denoted as 3layer, 3layer/3layer, and 5layer/5layer, were employed for the systematic aging and mechanical reliability evaluation. The individual Cu and Sn layer thicknesses were fixed at 1.5 μm and 2.5 μm, respectively. The layer sequences and nominal total thicknesses of Cu and Sn for each multilayer configuration are summarized in Table 2. The total interlayer thickness was calculated as the sum of the nominal Cu and Sn layer thicknesses deposited on the bonding surfaces, excluding the underlying Cu metallization and Ti adhesion layer.

Because the individual layer thicknesses were maintained constant, increasing the number of deposited layers simultaneously increased the number of Cu/Sn interfaces, the total Cu/Sn interlayer thickness, and the total amount of Cu and Sn within the bonding layer. Therefore, the present configurations represent distinct multilayer architectures rather than an isolated variation in the number of Cu/Sn interfaces at a constant total interlayer thickness. In addition, a 7layer/7layer configuration, fabricated using the same individual Cu and Sn layer thicknesses, was examined in the as-bonded state to further assess the microstructural consequences of increasing the multilayer architecture. Although the additional 7layer/7layer configuration extends the range of multilayer architectures examined, the number of Cu/Sn interfaces and the total interlayer thickness remain coupled variables because the individual layer thicknesses were fixed. Therefore, the independent effects of interface number and total interlayer thickness cannot be completely separated in the present study. The TLP bonding procedure is schematically illustrated in Figure 1b. The Si chip and DBC substrate were aligned and bonded according to the temperature–pressure profile shown in Figure 1c. Bonding was performed at a peak temperature of 280 °C under a constant pressure of 20 MPa in a vacuum atmosphere. After bonding, the specimens were allowed to cool naturally to room temperature. The interfacial microstructures were characterized using field-emission scanning electron microscopy (FE-SEM; Inspect F, FEI, Hillsboro, OR, USA) equipped with energy-dispersive X-ray spectroscopy (EDS; 300E, Thermo Fisher Scientific, Waltham, MA, USA). Quantitative EDS point analyses were performed at selected locations to identify the Cu-Sn intermetallic phases based on their Cu and Sn atomic compositions. To evaluate the thermal stability of the three primary configurations, isothermal aging was conducted at 175 °C for 0, 100, 300, and 500 h. The aging temperature of 175 °C was selected as an accelerated thermal aging condition rather than as a direct representation of the continuous operating temperature of the device. This temperature was employed to promote the Cu_6_Sn_5_ to Cu_3_Sn transformation and associated interfacial degradation within a practical experimental duration, thereby enabling comparison of the long-term microstructural stability of the three primary multilayer configurations. The thicknesses of the Cu_6_Sn_5_ and Cu_3_Sn IMC layers were evaluated from cross-sectional SEM images using ImageJ software (version 1.53e, National Institutes of Health, Bethesda, MD, USA). The IMC phases were identified based on SEM contrast and EDS compositional analysis, and their average thicknesses were calculated by dividing the corresponding cross-sectional areas by the analyzed interfacial length. Measurements were performed at least five randomly selected locations for each specimen, and the average values were reported.

The interfacial void fraction was quantified from cross-sectional SEM images using ImageJ software. The void fraction was calculated as the percentage of the total void area relative to the analyzed cross-sectional bonding area. Voids were identified and segmented based on the grayscale contrast between the void regions and the surrounding microstructure. Ten cross-sectional SEM images acquired at the same magnification were analyzed for each condition, with two images obtained from different regions of each of five independent bonded joints. The average values and corresponding standard deviations were calculated from the ten image-based measurements.

The mechanical reliability of the three primary configurations was evaluated using a die-shear tester (DAGE-4000, Nordson DAGE, UK), as illustrated in Figure 1d. The shear height and shear speed were fixed at 200 μm and 250 μm/s, respectively. The shear strength was calculated by dividing the maximum shear force by the nominal bonding area of 9 mm^2^ (3 × 3 mm^2^). Five specimens were tested for each condition, and the average values with the corresponding standard deviations were reported. Following the die-shear tests, the fracture surfaces were examined by FE-SEM and EDS to identify the fracture modes and correlate them with the corresponding interfacial microstructure.

## 3. Results and Discussion

**Figure 2:**
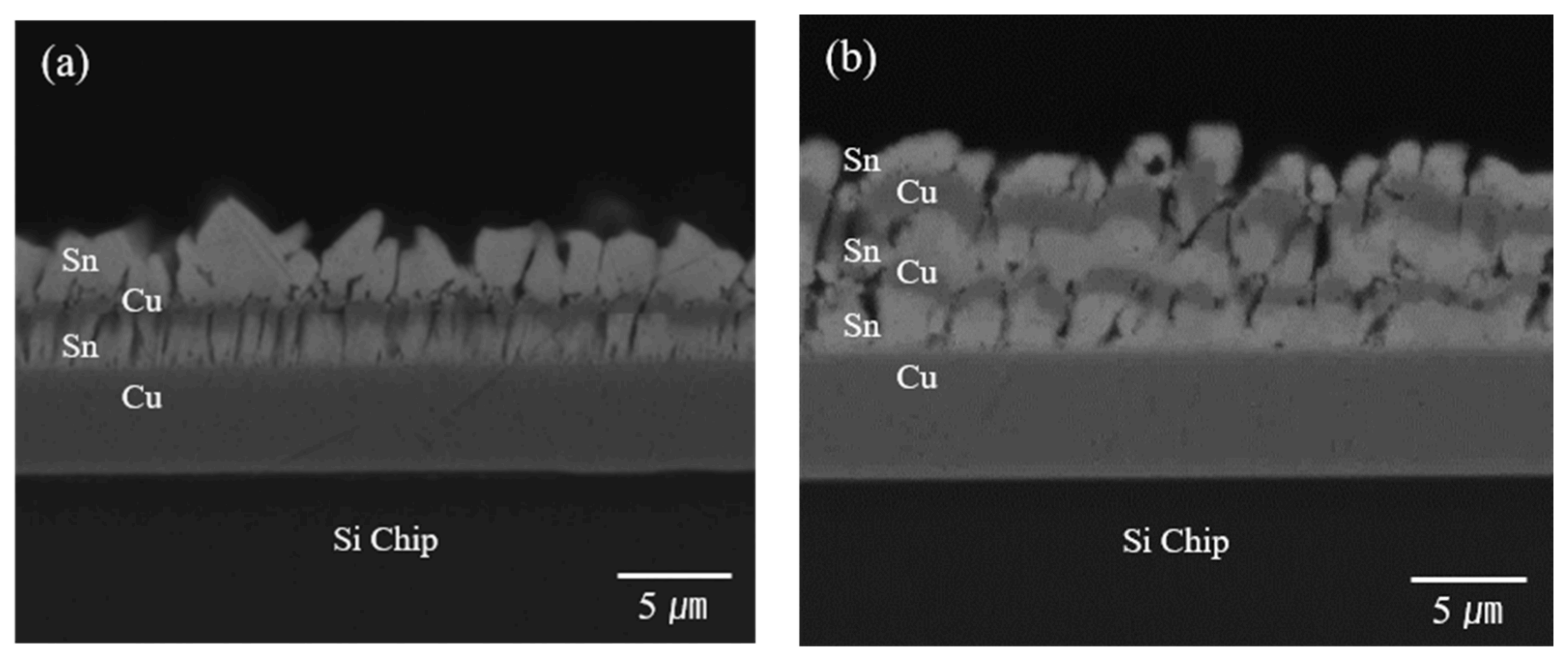
Cross-sectional SEM images of Cu/Sn multilayers deposited on the Si chip by sputtering: (**a**) 3layer and (**b**) 5layer structures.

Figure 2 presents cross-sectional SEM images of the Cu/Sn multilayers sputter-deposited on the Si chip prior to TLP bonding. Distinct alternating Cu and Sn layers were uniformly deposited, forming continuous multilayer structures with well-defined interfaces. No interfacial delamination, discontinuities, or other noticeable defects were observed, indicating that the sputtering process produced dense and structurally uniform multilayers. As the number of deposited Cu/Sn layers increased, the total multilayer thickness increased while maintaining a uniform layer morphology without significant interfacial defects. The combined changes in the number of Cu/Sn interfaces and total multilayer thickness are expected to modify the initial reaction pathway, the amount of material available for reaction, and the resulting IMC distribution during TLP bonding. The influence of these multilayer architectures on the subsequent Cu_6_Sn_5_ to Cu_3_Sn transformation during isothermal aging is discussed below.

**Figure 3:**
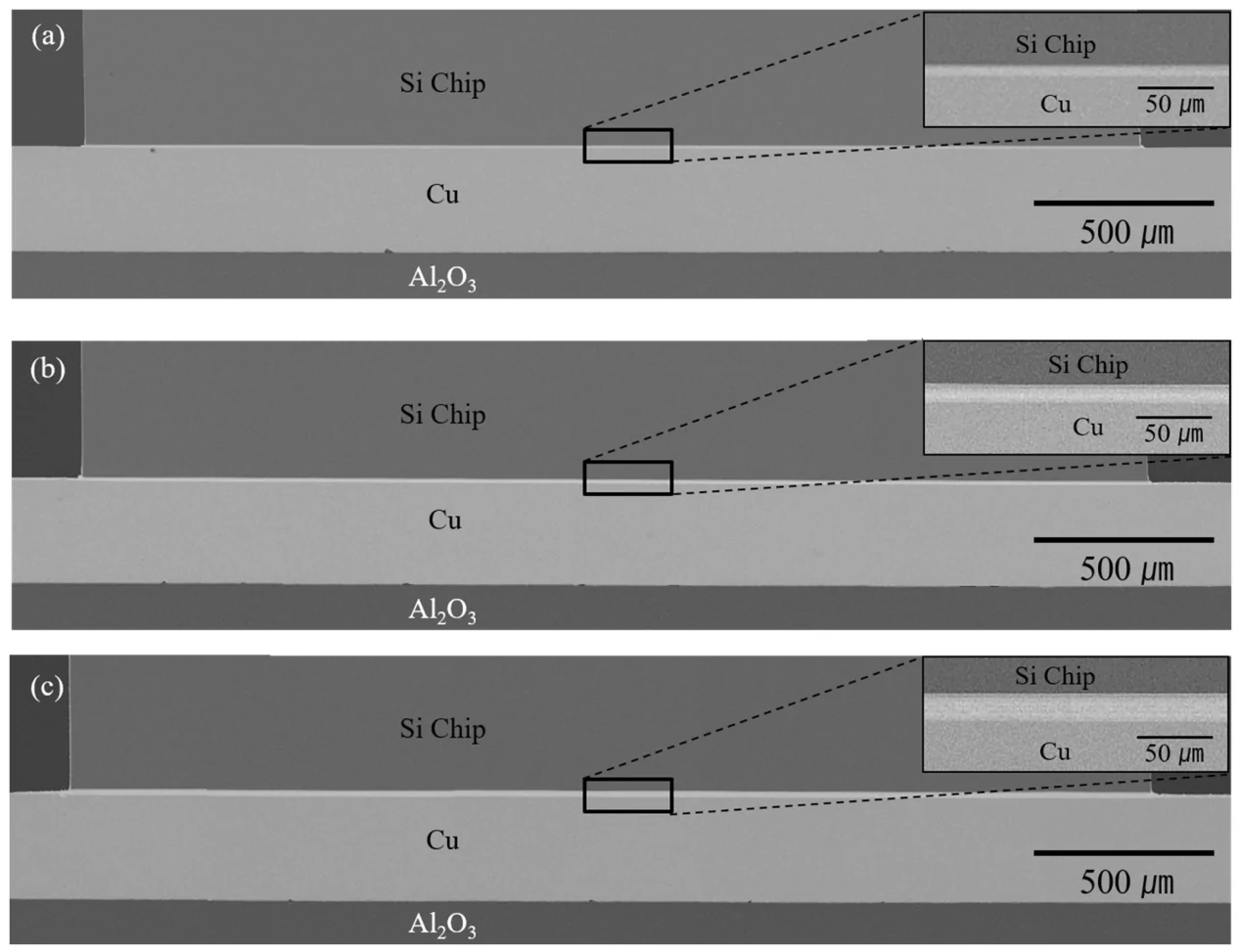
Cross-sectional SEM images of the as-bonded TLP joints fabricated with different Cu/Sn multilayer configurations: (**a**) 3layer, (**b**) 3layer/3layer, and (**c**) 5layer/5layer.

**Figure 4:**
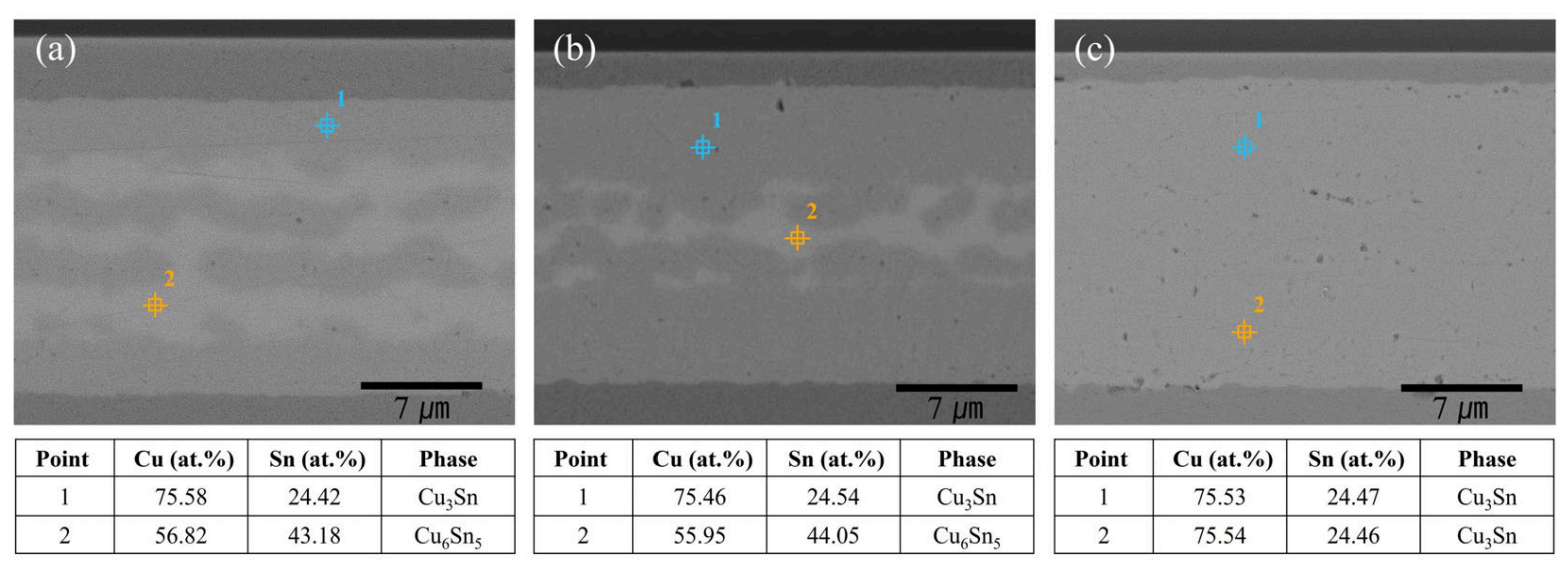
Representative cross-sectional SEM images and EDS point analyses of the 5layer/5layer TLP joint for phase identification: (**a**) as-bonded condition, (**b**) after 300 h, and (**c**) after 500 h of isothermal aging at 175 °C. The numbered points indicate the locations selected for quantitative EDS analysis, and the corresponding Cu and Sn atomic compositions and identified phases are summarized below each image.

Figure 3 presents the cross-sectional SEM images of the as-bonded TLP joints fabricated with different Cu/Sn multilayer configurations. Continuous TLP joints were successfully formed between the Si chip and the Cu substrate under all multilayer configurations. No apparent interfacial delamination, cracks, or noticeable voids were observed, indicating that sound interfacial bonding was achieved after the TLP bonding process. The DBC substrate used in this study was coated with OSP prior to bonding. OSP coatings are organic protective layers designed to suppress Cu oxidation during storage and handling. Previous studies have demonstrated thermal degradation of OSP coatings under typical lead-free soldering temperatures of 200–260 °C, with substantial degradation and disappearance of the OSP layer reported after thermal exposure at approximately 240 °C and above [29]. Therefore, during TLP bonding at 280 °C, substantial degradation and desorption of the OSP layer are expected, exposing the underlying Cu surface and enabling the interfacial reaction between Cu and molten Sn. The formation of Cu_6_Sn_5_ and Cu_3_Sn at the DBC side interface in the as-bonded specimens indicates that the OSP coating did not prevent the Cu-Sn reaction under the present bonding conditions. Nevertheless, because the decomposition and removal of the OSP layer were not directly characterized in this study, the possible influence of residual OSP derived species on the initial interfacial reaction cannot be completely excluded. Therefore, although the present microstructural observations indicate that OSP did not inhibit the formation and subsequent transformation of Cu-Sn IMCs, its potential secondary influence on the interfacial reaction should be considered when interpreting the results. The enlarged SEM images reveal that a thin and continuous reaction layer was uniformly formed along the bonding interface under all multilayer configurations. As the number of deposited Cu/Sn multilayers increased, the thickness of the reaction layer gradually increased while maintaining a continuous and defect-free interfacial morphology. The differences in the initial reaction-layer thickness and multilayer architecture are expected to influence the subsequent Cu_6_Sn_5_ to Cu_3_Sn transformation during isothermal aging. To further verify the phase identification based on the SEM contrast and morphology, quantitative EDS point analyses were performed, as shown in Figure 4.

To further verify the phase identification based on SEM contrast, representative EDS point analyses were performed on the 5layer/5layer joint in the as-bonded condition and after 300 and 500 h of isothermal aging, as shown in Figure 4. In the as-bonded condition, the Cu and Sn compositions were 75.58 and 24.42 at.% at Point 1 and 56.82 and 43.18 at.% at Point 2, respectively, corresponding to Cu_3_Sn and Cu_6_Sn_5_. After 300 h of aging, the EDS results confirmed the coexistence of Cu_6_Sn_5_ and Cu_3_Sn, indicating that the phase transformation was still in progress. After 500 h of aging, both analyzed regions exhibited compositions of approximately 75.5 at.% Cu and 24.5 at.% Sn, consistent with Cu_3_Sn. These EDS results support the phase assignments based on SEM contrast and further confirm the transformation of the Cu_6_Sn_5_-rich region into Cu_3_Sn during prolonged aging.

**Figure 5:**
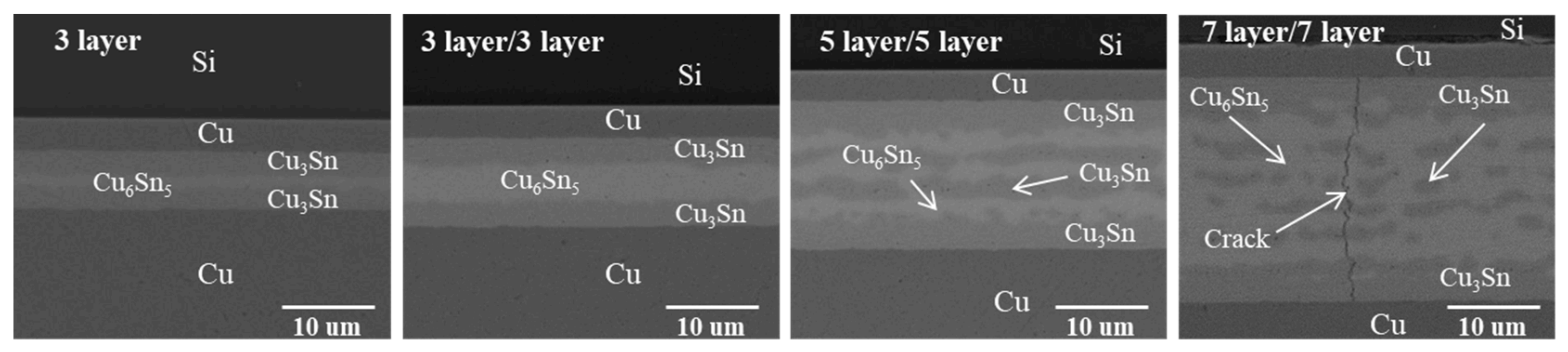
Cross-sectional SEM images of the as-bonded TLP joints with different Cu/Sn multilayer configurations: 3layer, 3layer/3layer, 5layer/5layer, and 7layer/7layer.

Figure 5 compares the as-bonded microstructures of the TLP joints with different Cu/Sn multilayer configurations, including the additional 7layer/7layer configuration. The 3layer, 3layer/3layer, and 5layer/5layer joints exhibited Cu_6_Sn_5_ and Cu_3_Sn phases with configuration-dependent distributions, consistent with the phase identification described above. In contrast, localized cracking was observed within the bonding layer of the 7layer/7layer configuration. Similar localized cracks were observed in the examined 7layer/7layer joints, although they were confined to limited regions and were not distributed throughout the entire bonding area. The presence of localized cracking in the as-bonded state indicates that further increasing the multilayer architecture may introduce additional microstructural complications during TLP bonding. However, because the number of Cu/Sn interfaces and the total interlayer thickness increase simultaneously in the present architecture, the observed cracking cannot be attributed solely to the increased number of interfaces. Previous studies have reported stress evolution during reactive interdiffusion in Cu-Sn systems [30] and the brittle fracture characteristics of Cu_3_Sn and Cu_6_Sn_5_ intermetallic compounds [31]. These findings indicate that the mechanical integrity of the increasingly complex multilayer structure should also be considered when increasing the number of Cu/Sn layers. However, because the stress state and crack-driving force were not directly measured in the present study, the origin of the observed cracking cannot be conclusively determined.

**Figure 6:**
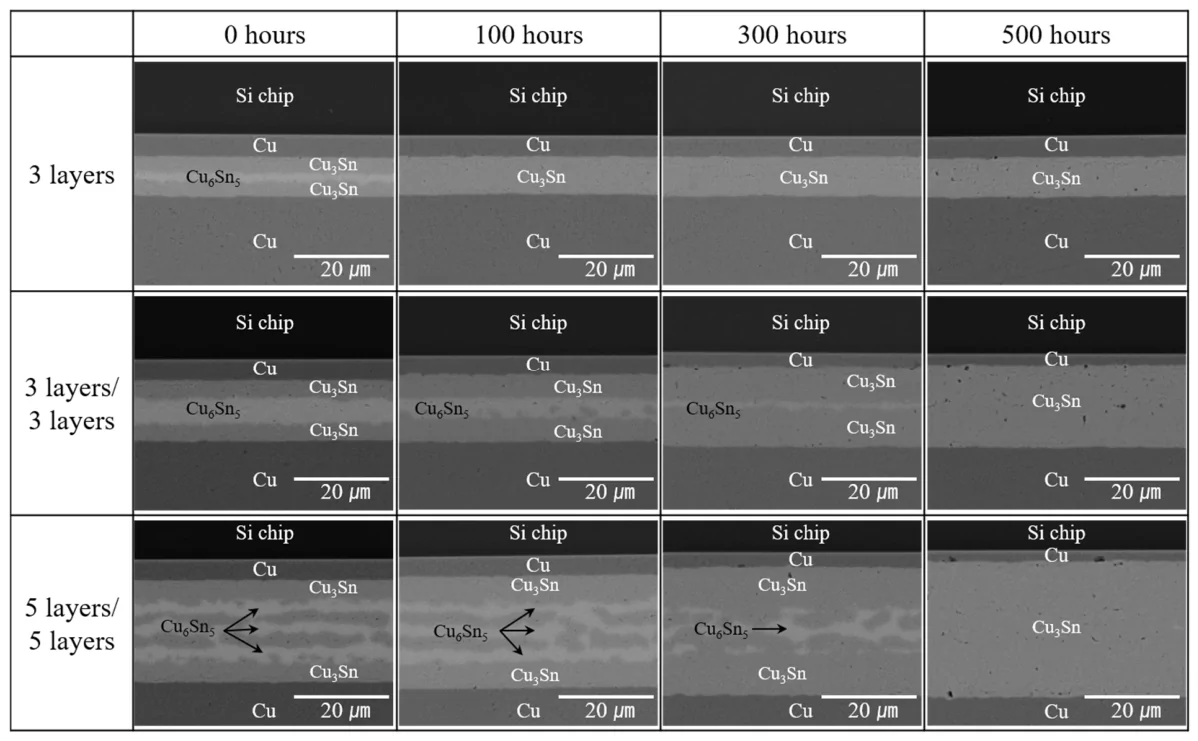
Cross-sectional SEM images showing the interfacial microstructural evolution of the TLP joints during isothermal aging at 175 °C under different Cu/Sn multilayer configurations.

Figure 6 presents the microstructural evolution of the three primary TLP joint configurations during isothermal aging at 175 °C for up to 500 h, with the as-bonded microstructures included as the initial reference. The interfacial microstructure progressively evolved with increasing aging time through the transformation of Cu_6_Sn_5_ into Cu_3_Sn, followed by further growth of the Cu_3_Sn layer. This transformation behavior was strongly influenced by the initial Cu/Sn multilayer architecture. For the 3layer specimen, the as-bonded joint consisted of a thin Cu_6_Sn_5_ layer and a relatively thick Cu_3_Sn layer. During isothermal aging, the remaining Cu_6_Sn_5_ was completely transformed into Cu_3_Sn after 100 h, after which the Cu_3_Sn layer remained stable up to 500 h. In contrast, the 3layer/3layer specimen initially exhibited Cu_6_Sn_5_ and Cu_3_Sn layers with comparable thicknesses. The Cu_6_Sn_5_ layer gradually decreased during aging, remained detectable up to 300 h, and disappeared only after 500 h. A similar but more pronounced trend was observed for the 5layer/5layer specimen. In the as-bonded condition, Cu_3_Sn layers had already formed adjacent to both the Si chip and the DBC substrate, whereas a Cu_6_Sn_5_-rich region containing island-shaped Cu_3_Sn remained in the center of the bonding layer. This intermediate microstructure persisted up to 300 h, and complete transformation into a continuous Cu_3_Sn layer was achieved only after 500 h. These observations suggest that the initial Cu/Sn multilayer architecture significantly influenced the transformation kinetics of Cu_6_Sn_5_ into Cu_3_Sn. The 3layer configuration exhibited the most rapid completion of the transformation, which can be associated with its thinner overall interlayer and the smaller amount of residual Cu_6_Sn_5_ requiring subsequent transformation. In contrast, the thicker multilayer configurations exhibited a prolonged transformation process, as the larger amount of residual Cu_6_Sn_5_-rich regions had to be progressively consumed through interdiffusion before a continuous Cu_3_Sn layer could be established throughout the bonding layer. Consequently, specimens with thicker multilayer configurations, which contained both a larger number of Cu/Sn interfaces and a greater total amount of deposited Cu and Sn, required a longer aging time to complete the Cu_6_Sn_5_ to Cu_3_Sn transformation. As the aging time increased, both the number and size of microvoids gradually increased, particularly in the specimens with thicker multilayer configurations. The origin of the observed voids appeared to depend on their location within the bonding layer. The voids observed near the Si chip may be associated with the Kirkendall effect arising from the difference in the diffusion rates of Cu and Sn during Cu_3_Sn growth. However, the possible contribution of pre-existing defects in the sputtered Cu/Sn multilayers to void formation cannot be excluded. Previous studies have reported that Kirkendall voids preferentially develop in electroplated or electroless Cu rather than in high-purity Cu because the higher defect density facilitates vacancy accumulation [32,33]. Since electroplated Cu was used on the Si chip, whereas the DBC substrate consisted of high-purity Cu, the preferential formation of voids near the Si chip is consistent with this mechanism.

In contrast, the voids observed in the center of the bonding layer are considered to originate from the combined effects of the Cu_6_Sn_5_ to Cu_3_Sn transformation, diffusion-induced vacancy accumulation, and the initial Cu/Sn multilayer architecture introduced during sputtering. In the 5layer/5layer specimen after 500 h, the central voids were located at positions corresponding to the initial Cu_6_Sn_5_-rich region, suggesting that the gradual consumption of these regions during Cu_3_Sn formation promoted localized vacancy accumulation. Furthermore, differences in the initial multilayer configurations, including the number of Cu/Sn interfaces and the total amount of deposited material, may have influenced vacancy condensation during prolonged interdiffusion, thereby affecting void nucleation and subsequent growth. Because the diffusion coefficient of Cu in Cu_3_Sn has been reported to be approximately 7 times higher than that of Sn at 423 K, a temperature close to the isothermal aging condition employed in this study (175 °C), the resulting imbalance in atomic flux can promote vacancy supersaturation during prolonged interdiffusion, thereby facilitating Kirkendall void formation [34]. The configuration-dependent evolution of these interfacial voids was further evaluated quantitatively by measuring the interfacial void fraction. These observations indicate that the long-term microstructural evolution of the TLP joints is influenced by both diffusion-induced vacancy accumulation during Cu_3_Sn growth and the initial Cu/Sn multilayer architecture.

**Figure 7:**
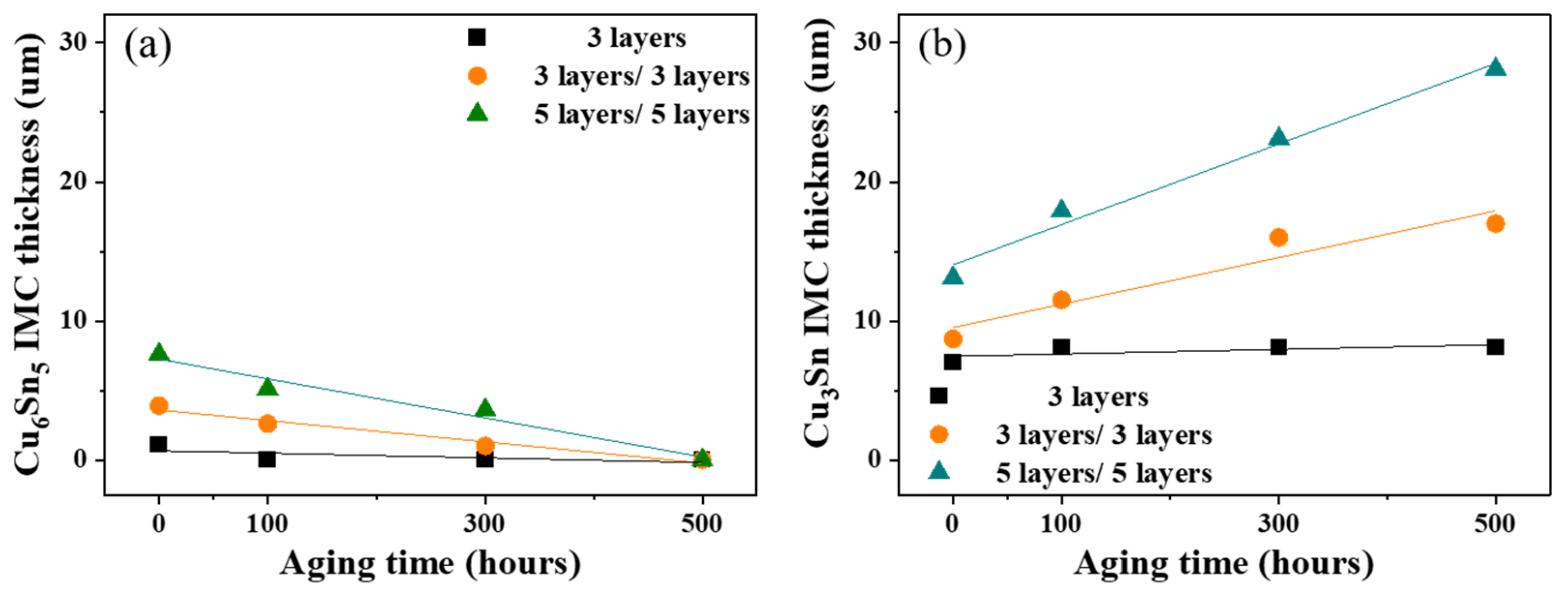
Thickness evolution of (**a**) Cu_6_Sn_5_ and (**b**) Cu_3_Sn IMCs during isothermal aging under different Cu/Sn multilayer configurations.

**Figure 8:**
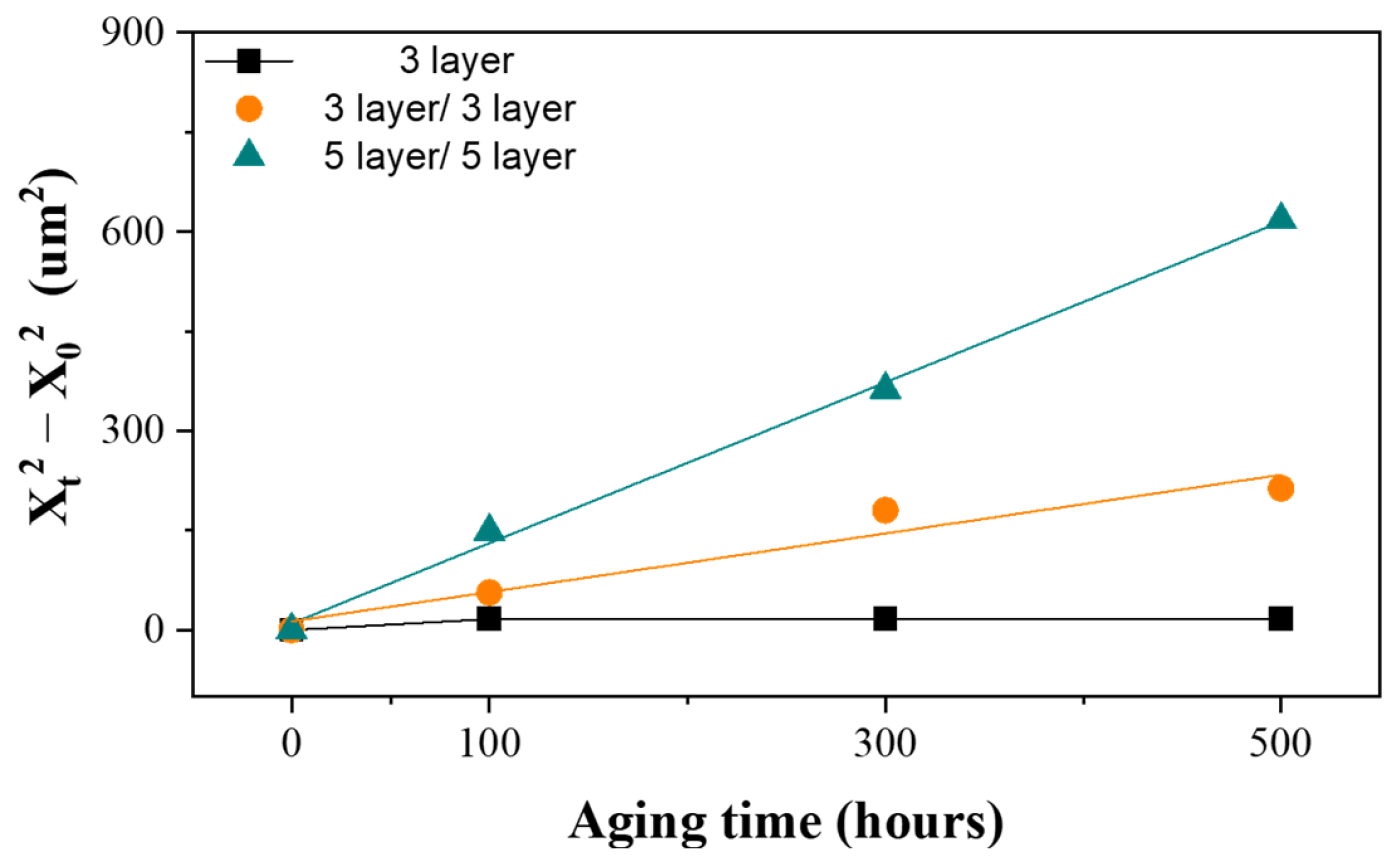
Apparent Cu_3_Sn growth kinetics during isothermal aging at 175 °C under different Cu/Sn multilayer configurations. Solid lines represent linear fits for the 3layer/3layer and 5layer/5layer configurations. The 3layer configuration was not fitted because Cu_6_Sn_5_ was completely consumed within the first 100 h of aging, resulting in negligible subsequent Cu_3_Sn growth.

The microstructural evolution shown in Figure 6 was further quantified by measuring the thicknesses of the Cu_6_Sn_5_ and Cu_3_Sn layers, as summarized in Figure 7. The Cu_6_Sn_5_ layer gradually decreased with increasing aging time under all multilayer configurations, whereas the Cu_3_Sn layer continuously increased because of the progressive transformation of Cu_6_Sn_5_ into Cu_3_Sn during isothermal aging. Under the 3layer condition, the Cu_6_Sn_5_ layer disappeared after 100 h, indicating that the transformation of Cu_6_Sn_5_ into Cu_3_Sn was completed during the early stage of aging. In contrast, under the 3layer/3layer and 5layer/5layer conditions, the Cu_6_Sn_5_ layer remained detectable up to 300 h and was completely transformed into Cu_3_Sn only after 500 h. These quantitative results are in good agreement with the SEM observations presented in Figure 6. The Cu_3_Sn layer was thicker in the configurations with a greater number of deposited Cu/Sn multilayers and a correspondingly greater total interlayer thickness. The 5layer/5layer specimen exhibited the greatest Cu_3_Sn thickness throughout the aging period, which can be attributed to its different initial multilayer architecture, including both the larger number of Cu/Sn interfaces and the greater total amount of deposited Cu and Sn. These differences prolonged the overall transformation process and allowed continued Cu_3_Sn formation during prolonged aging. Consequently, the transformation from Cu_6_Sn_5_ to Cu_3_Sn proceeded over a longer period, resulting in a thicker Cu_3_Sn layer after complete transformation. These differences in Cu_3_Sn evolution indicate that the initial multilayer configuration affects not only the final IMC thickness but also the duration and apparent kinetics of the Cu_6_Sn_5_ to Cu_3_Sn transformation. Therefore, the Cu_3_Sn growth behavior was further quantitatively evaluated using a kinetic analysis, as shown in Figure 8.

To provide a comparative assessment of the apparent Cu_3_Sn growth behavior among the different multilayer configurations, the thickness evolution was analyzed using the parabolic growth relationship, which is commonly employed to describe diffusion-controlled intermetallic compound growth [35,36].

$$X_{t}^{2}-X_{0}^{2}=Kt$$

where $X_{t}$ and $X_{0}$ represent the Cu_3_Sn thicknesses after aging for time t and in the as-bonded condition, respectively, and K is the apparent growth rate constant. Figure 8 presents $X_{t}^{2}-X_{0}^{2}$ as a function of aging time for the different Cu/Sn multilayer configurations. The apparent growth rate constants were obtained from linear regression without constraining the intercept to zero. The 3layer/3layer and 5layer/5layer joints exhibited approximately linear relationships, with R^2^ values of approximately 0.941 and 0.998, respectively. These trends are broadly consistent with diffusion-controlled growth behavior; however, the relatively high R^2^ values should not be regarded as definitive validation of a parabolic growth mechanism because the regression was based on a limited number of aging time points at a single temperature. The corresponding apparent growth rate constants were approximately 0.44 ± 0.08 and 1.21 ± 0.04 μm^2^/h (standard errors of the fitted slopes) for the 3layer/3layer and 5layer/5layer configurations, respectively.

In contrast, the 3layer joint was not subjected to linear fitting because the remaining Cu_6_Sn_5_ was completely consumed within the first 100 h of aging. Consequently, the Cu_3_Sn thickness increased from approximately 7.0 to 8.1 μm during the initial aging stage and subsequently remained nearly constant up to 500 h. This behavior reflects the early completion of the Cu_6_Sn_5_ to Cu_3_Sn transformation and the limited amount of remaining reactant available for further Cu_3_Sn growth, rather than continuous Cu_3_Sn growth throughout the entire aging period. In contrast, the 3layer/3layer and 5layer/5layer joints retained Cu_6_Sn_5_ for longer aging periods, allowing continued Cu_3_Sn formation through solid-state interdiffusion.

The higher apparent growth rate observed for the 5layer/5layer configuration should therefore be interpreted in relation to the initial multilayer architecture and the larger amount of Cu_6_Sn_5_ available for subsequent transformation, rather than solely as an intrinsic increase in the diffusivity of Cu or Sn. These results suggest that the apparent Cu_3_Sn growth behavior may be influenced by the initial Cu/Sn multilayer configuration, particularly through differences in the amount of Cu_6_Sn_5_ available for subsequent transformation. Accordingly, the apparent growth constants obtained in the present study should be regarded as comparative parameters describing the configuration-dependent transformation behavior rather than intrinsic diffusion coefficients. Further studies employing additional aging temperatures and shorter aging intervals would be useful for establishing a more rigorous kinetic description of Cu_3_Sn growth.

**Figure 9:**
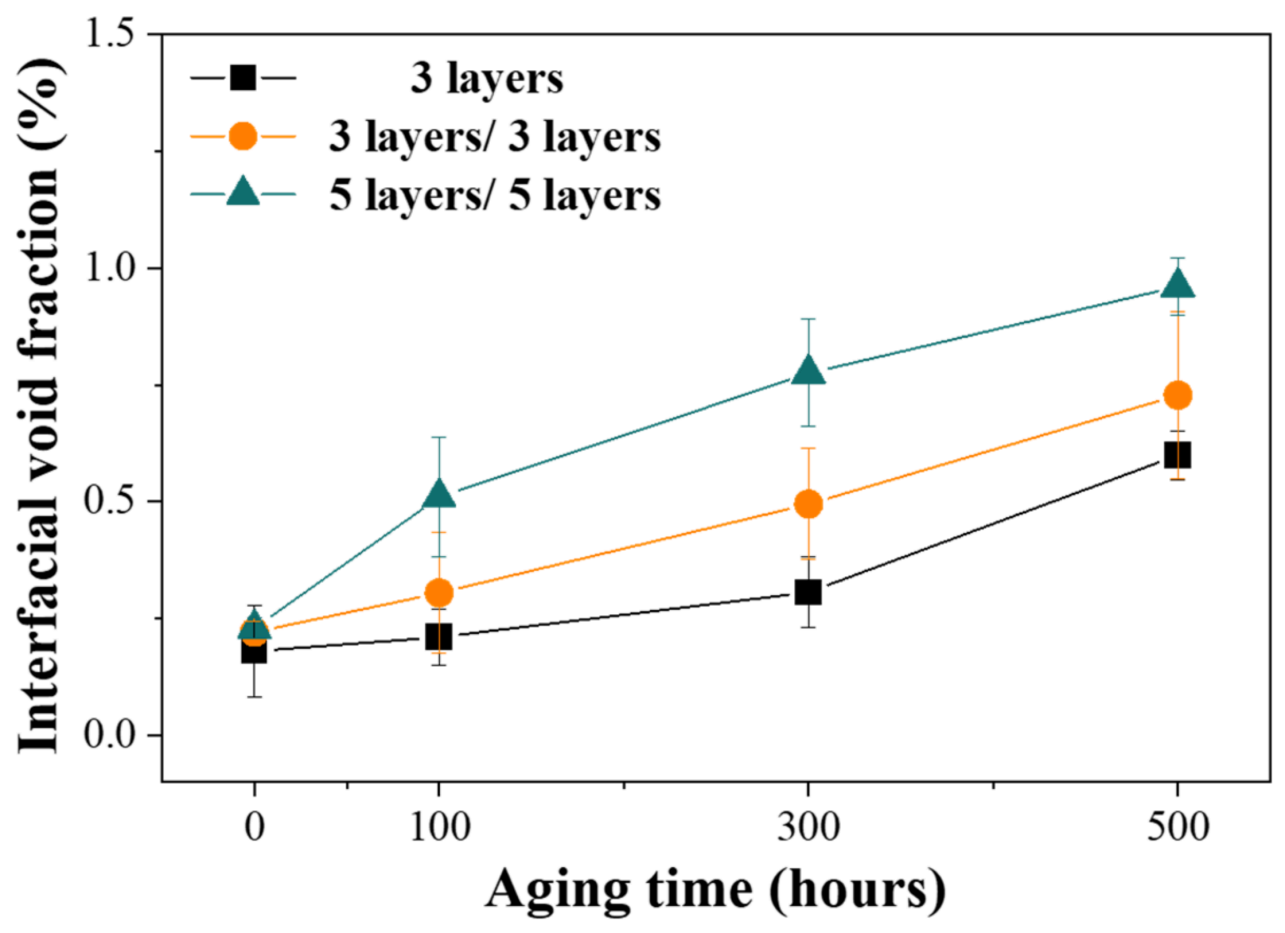
Interfacial void fraction of the TLP joints as a function of isothermal aging time at 175 °C under different Cu/Sn multilayer configurations.

Figure 9 shows the evolution of the interfacial void fraction in the TLP joints during isothermal aging at 175 °C. In the as-bonded condition, all three multilayer configurations exhibited low interfacial void fractions. With increasing aging time, the interfacial void fraction showed an increasing trend for all joints. Among the three configurations, the 5layer/5layer joint generally exhibited the highest void fraction during aging, whereas the differences between the 3layer and 3layer/3layer joints were relatively small at the intermediate aging times. After 500 h, the interfacial void fractions reached approximately 0.60%, 0.73%, and 0.96% for the 3layer, 3layer/3layer, and 5layer/5layer joints, respectively. These results quantitatively support the progressive development of interfacial voids observed in the cross-sectional microstructures shown in Figure 6. The greater void accumulation observed in the 5layer/5layer joint may be associated with differences in the initial multilayer structures and material quantities during aging. Such void accumulation can locally reduce the effective load-bearing area and facilitate crack initiation, thereby potentially contributing to the mechanical degradation observed after prolonged aging.

**Figure 10:**
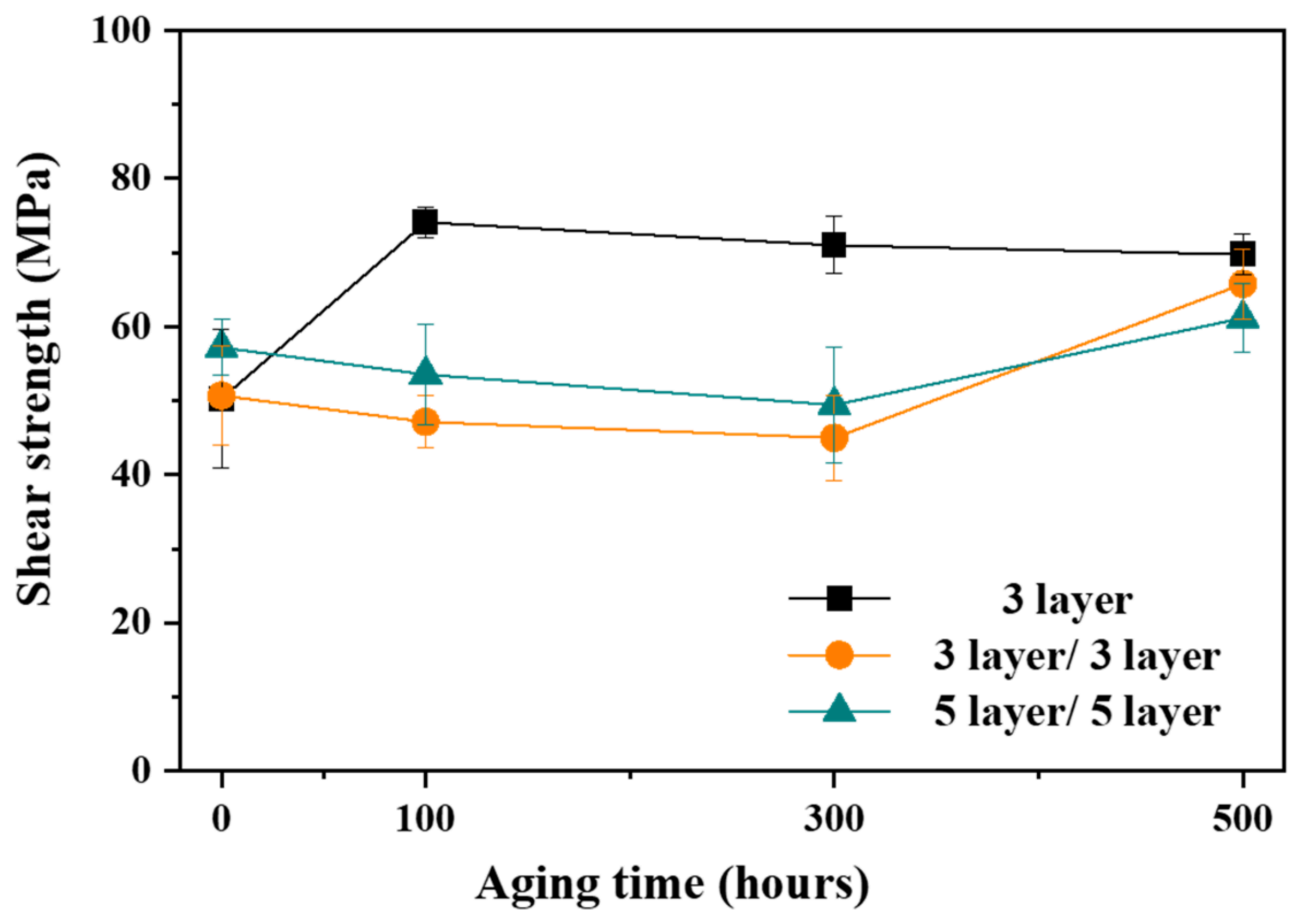
Shear strength of the TLP joints after isothermal aging under different Cu/Sn multilayer configurations.

Figure 10 presents the evolution of shear strength in the TLP joints during isothermal aging under different Cu/Sn multilayer configurations. The shear strength evolution was strongly influenced by the interfacial microstructure established during bonding and its subsequent evolution during isothermal aging. The 3layer specimen exhibited the lowest initial shear strength because the joint had already transformed predominantly into Cu_3_Sn during bonding while retaining a thin Cu_6_Sn_5_ layer at the interface. After 100 h of aging, the remaining Cu_6_Sn_5_ was completely transformed into Cu_3_Sn, resulting in an approximately 47% increase in shear strength. This improvement may be associated with the elimination of the Cu_6_Sn_5_/Cu_3_Sn interface, which has been reported to be more susceptible to crack initiation than a fully developed Cu_3_Sn joint during mechanical loading [37,38,39,40,41]. Once the interfacial reaction was completed, the shear strength gradually decreased with further aging because the beneficial effect of complete Cu_3_Sn formation was progressively offset by the development of interfacial voids.

**Figure 11:**
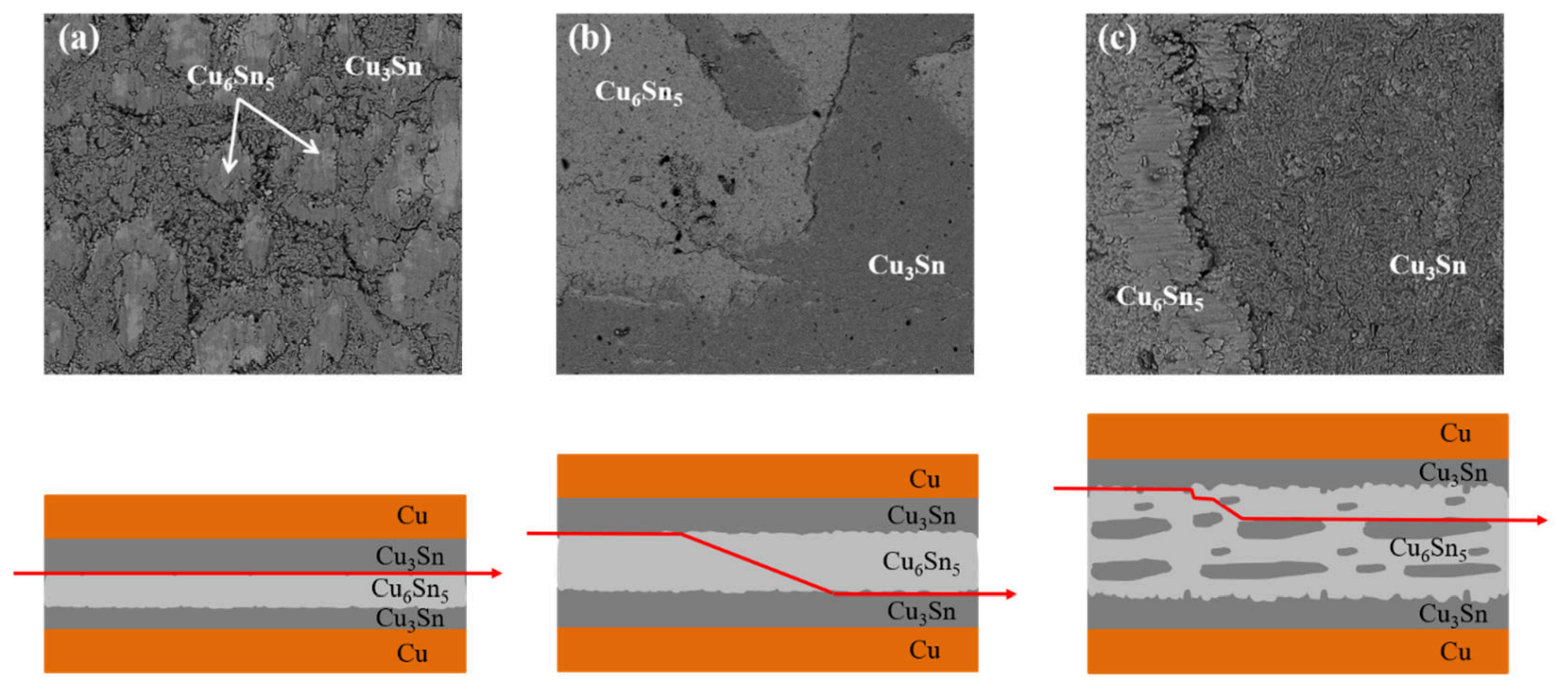
FE-SEM images of the fracture surfaces of the as-bonded TLP joints under different Cu/Sn multilayer configurations: (**a**) 3layer, (**b**) 3layer/3layer, and (**c**) 5layer/5layer. The red arrows in the schematic illustrations indicate the fracture paths.

In contrast, the 3layer/3layer and 5layer/5layer specimens retained Cu_6_Sn_5_ for a considerably longer period, which can be attributed to differences in their initial multilayer architectures, including both the larger number of Cu/Sn interfaces and the greater total amount of deposited Cu and Sn. Consistent with the microstructural evolution described in Figure 6, the delayed transformation into a continuous Cu_3_Sn layer may have contributed to relatively lower shear strengths during the early stage of aging. After 500 h, the remaining Cu_6_Sn_5_ was completely transformed into Cu_3_Sn, leading to an increase in shear strength in both specimens. However, their final shear strengths remained lower than that of the 3layer specimen, which is consistent with the greater interfacial void accumulation observed after prolonged aging, as quantitatively demonstrated by the void-fraction results in Figure 9. In particular, although the 5layer/5layer specimen exhibited the thickest Cu_3_Sn layer after complete transformation, the strengthening effect associated with the formation of a continuous Cu_3_Sn joint was partially offset by its higher interfacial void fraction. These observations suggest that the mechanical performance of the TLP joints is governed not only by the extent of Cu_3_Sn formation but also by the degree of interfacial void development during aging. These findings demonstrate that the mechanical reliability of Cu/Sn multilayer TLP joints is determined by the balance between the beneficial effect of Cu_6_Sn_5_ to Cu_3_Sn transformation and the detrimental effect of interfacial void evolution during prolonged aging. Complete transformation into Cu_3_Sn improves the mechanical integrity of the bonding layer by eliminating the mechanically weak Cu_6_Sn_5_/Cu_3_Sn interface, whereas excessive interfacial void formation progressively reduces the load-bearing capability of the joint. Optimizing the initial Cu/Sn multilayer architecture to promote rapid Cu_3_Sn formation while suppressing excessive void development is therefore essential for achieving high long-term mechanical reliability. These findings are consistent with previous studies on TLP and sintered die-attach joints, which have similarly reported that long-term mechanical reliability is governed by the balance between interfacial reaction kinetics and void evolution during thermal aging [42]. To further clarify the relationship between shear strength and fracture behavior, the fracture surfaces of the as-bonded TLP joints after the die-shear test were examined, as presented in Figure 11.

To further clarify the fracture mechanisms underlying the differences in shear strength shown in Figure 10, the fracture surfaces of the as-bonded TLP joints were examined by FE-SEM, as presented in Figure 11. Under the 3layer condition (Figure 11a), fracture occurred predominantly along the Cu_6_Sn_5_/Cu_3_Sn interface, indicating that crack propagation preferentially followed the Cu_6_Sn_5_/Cu_3_Sn interface. This observation is consistent with previous studies reporting that the Cu_6_Sn_5_/Cu_3_Sn interface is susceptible to crack initiation because of the mismatch in the coefficient of thermal expansion (CTE) and the mechanical incompatibility between the two IMCs [43]. The 3layer/3layer joint (Figure 11b) exhibited a similar fracture behavior, with crack propagation occurring mainly along the Cu_6_Sn_5_/Cu_3_Sn interface. Compared with the 3layer joint, the thicker Cu_6_Sn_5_ layer resulted in a longer fracture path through the mixed IMC structure. The 5layer/5layer joint (Figure 11c) exhibited a more tortuous fracture path because the remaining Cu_6_Sn_5_-rich region containing island-shaped Cu_3_Sn led to a more heterogeneous interfacial microstructure. The corresponding schematic illustrations shown beneath each FE-SEM image agree well with the observed fracture morphologies. These observations demonstrate that the fracture path was strongly governed by the initial interfacial IMC structure, which is consistent with the variation in the initial shear strength shown in Figure 10.

The superior initial shear strength of the 5layer/5layer joint can be further understood in terms of the mechanical role of the island-shaped Cu_3_Sn region embedded within the Cu_6_Sn_5_-rich matrix. In the 3layer and 3layer/3layer joints, the Cu_6_Sn_5_ phase formed a continuous, planar layer along the bonding interface, providing a single, mechanically weak crack path with minimal resistance to propagation. In contrast, the discontinuous, island-like distribution of Cu_3_Sn within the 5layer/5layer joint interrupted this planar weak interface. This possible crack-deflection mechanism is analogous to the toughening mechanisms observed in particle-reinforced composite systems, where a dispersed secondary phase embedded in a continuous matrix impedes straightforward crack propagation. In this case, the harder and more mechanically robust Cu_3_Sn islands may have acted as local microstructural barriers to crack propagation within the relatively weaker Cu_6_Sn_5_-matrix, deflecting crack propagation and potentially promoting a more distributed fracture path across the bonding layer, thereby suppressing localized stress concentration at the interface. Consequently, the discontinuous, island-shaped Cu_3_Sn morphology-rather than a continuous Cu_6_Sn_5_ interfacial layer-may have hindered crack propagation, contributing to the highest initial shear strength among the three configurations.

**Figure 12:**
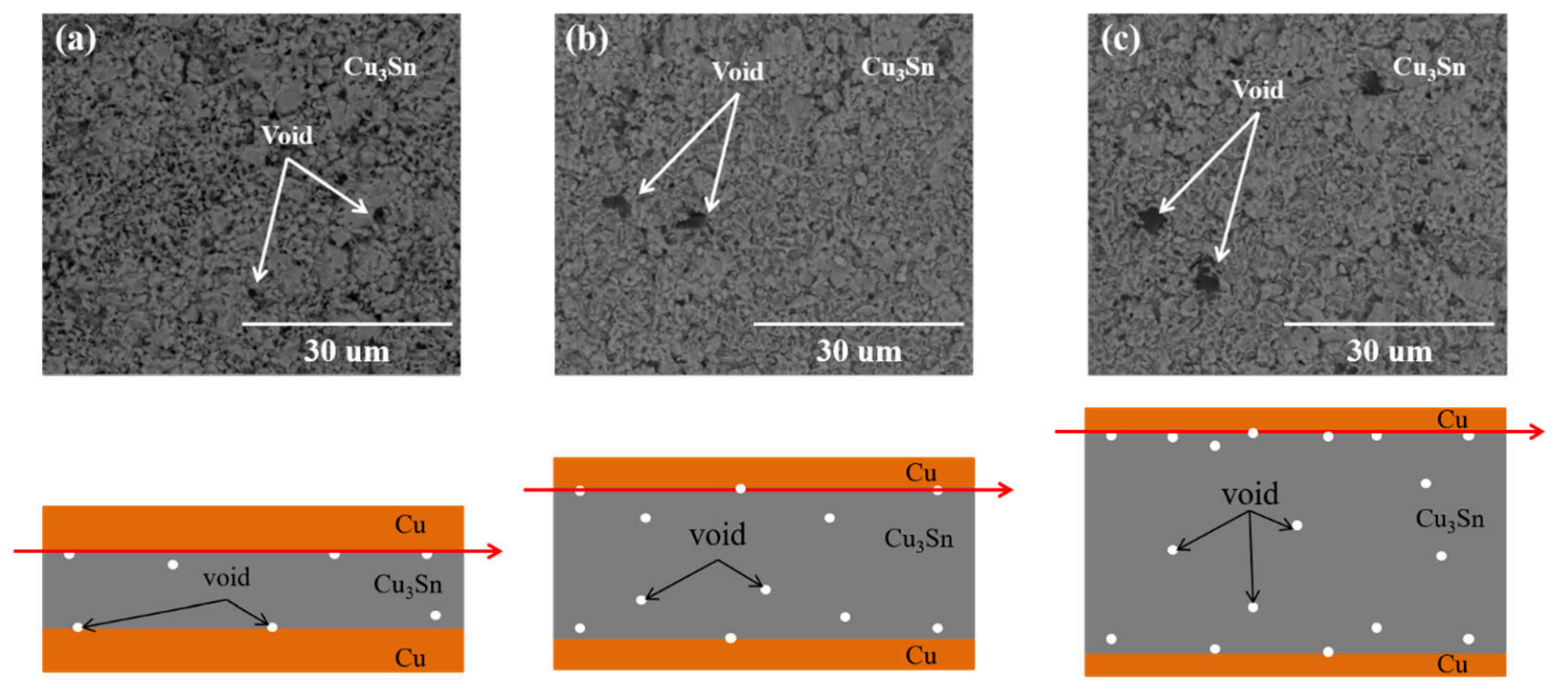
FE-SEM images of the fracture surfaces of the TLP joints after 500 h of isothermal aging under different Cu/Sn multilayer configurations: (**a**) 3layer, (**b**) 3layer/3layer, and (**c**) 5layer/5layer. The white arrows indicate voids, while the red arrows in the schematic illustrations indicate the fracture paths.

Figure 12 presents the fracture surfaces of the TLP joints after 500 h of isothermal aging. After prolonged aging, fracture occurred predominantly along the Cu_3_Sn/Cu interface for all multilayer configurations, in contrast to the fracture behavior observed in the as-bonded joints shown in Figure 11. Interfacial voids were observed in all specimens after prolonged aging, consistent with the qualitative microstructural observations in Figure 6 and the quantitative interfacial void-fraction results in Figure 9. The 3layer joint (Figure 12a) exhibited relatively few and small voids, whereas both the size and number of voids increased markedly in the 3layer/3layer and 5layer/5layer joints (Figure 12b,c). The corresponding schematic illustrations are in good agreement with the observed fracture morphologies and void distributions. The progressive increase in interfacial void fraction during prolonged aging is consistent with the reduction in shear strength shown in Figure 10, suggesting that progressive void evolution may have contributed to the reduction in the effective load-bearing area of the bonding layer, although the shear strength was calculated using the nominal bonding area. These observations are also consistent with previous studies reporting that interfacial void formation, including Kirkendall voids, can degrade the mechanical performance of Cu3Sn joints during thermal aging [44,45]. From an engineering perspective, these findings suggest that the simplified 3layer configuration exhibited better mechanical performance under the investigated aging conditions, which may be associated with the formation of a continuous Cu_3_Sn bonding layer and relatively limited interfacial void development during aging. The thicker Cu/Sn multilayer architectures contained both a greater number of interfaces and a larger amount of deposited Cu and Sn. These configurations exhibited delayed Cu_6_Sn_5_ to Cu_3_Sn transformation and greater void accumulation during aging, which may have offset the mechanical benefit of complete Cu_3_Sn formation. These results suggest that the overall multilayer architecture, rather than the number of Cu/Sn interfaces alone, influences the microstructural evolution and mechanical performance of TLP joints. Therefore, optimizing the initial Cu/Sn multilayer architecture to balance interfacial reaction kinetics and void evolution is important for improving the mechanical reliability of TLP joints.

The present study evaluated mechanical reliability using a single die-shear configuration and isothermal aging at 175 °C for up to 500 h. Therefore, the mechanical behavior at other operating temperatures and the long-term reliability beyond the investigated aging period remain to be verified. In addition, thermal cycling and electrical reliability, which are critical for power module applications, were beyond the scope of this study and should be investigated in future work. Future studies incorporating these evaluations, together with finite element analysis to quantify the thermomechanical stress distribution arising from CTE mismatch among the Si chip, Cu-Sn intermetallic bonding layer, and DBC substrate-particularly the stress concentration near critical IMC interfaces and around interfacial voids-would further clarify the relationship between microstructural evolution and long-term mechanical reliability under realistic thermal cycling conditions.

## 4. Conclusions

In this study, a sputtered Cu/Sn multilayer TLP bonding process was developed to investigate the effect of the initial Cu/Sn multilayer configuration on the interfacial microstructure and mechanical performance of TLP joints during isothermal aging at 175 °C for up to 500 h. Three primary configurations, namely 3layer, 3layer/3layer, and 5layer/5layer, were systematically evaluated, while an additional 7layer/7layer configuration was examined in the as-bonded state. Localized cracking observed in the 7layer/7layer joints indicated potential microstructural complications associated with further increasing the multilayer architecture. In the three primary configurations, Cu_6_Sn_5_ and Cu_3_Sn IMCs coexisted within the bonding layer in the as-bonded state, whereas all three joints were completely transformed into Cu_3_Sn after 500 h of isothermal aging at 175 °C. The interfacial evolution strongly depended on the initial Cu/Sn multilayer configuration, which influenced IMC transformation, interfacial void development, and shear strength. A comparative kinetic analysis suggested that the 5layer/5layer configuration exhibited a higher apparent Cu_3_Sn growth rate, which may reflect differences in the initial multilayer architecture and the amount of Cu_6_Sn_5_ available for subsequent transformation. The interfacial void fraction increased with aging time for all three primary configurations, with the 5layer/5layer joint exhibiting the greatest void accumulation after 500 h. Although the Cu_6_Sn_5_ to Cu_3_Sn transformation initially improved shear strength, progressive void accumulation during prolonged aging contributed to subsequent mechanical degradation. Fracture paths evolved from the initial IMC-dependent interfaces to predominantly the Cu_3_Sn/Cu interface after prolonged aging. From an application perspective, these results indicate that simply increasing the number of Cu/Sn layers is not necessarily advantageous for high-temperature die-attach applications. Although the 5layer/5layer configuration provided high initial shear strength, its greater void accumulation during isothermal aging was associated with a reduction in its mechanical advantage after 500 h. In contrast, the 3layer configuration achieved earlier completion of the Cu_6_Sn_5_ to Cu_3_Sn transformation and maintained the highest shear strength after 500 h. Therefore, for high-temperature power electronic packaging, multilayer design should prioritize rapid establishment of a Cu_3_Sn joint while minimizing subsequent interfacial void accumulation, rather than maximizing the number of Cu/Sn layers.

## Figures and Tables

**Table 1:** Sputtering process parameters for Cu/Sn multilayer deposition.

Process	Target	Gas	Base Pressure (Torr)	Working Pressure (Torr)	Time (min)	DC Current (A)	Power (W)	Deposition Rate (nm/min)
**Pre-cleaning**	–	Ar	≤1 × 10^−5^	3.6 × 10^−3^	15	0.3	30	–
**Pre-sputtering**	–	Ar	≤1 × 10^−5^	3.6 × 10^−3^	10	0.3	50	–
**Sputtering**	Sn	Ar	≤1 × 10^−5^	3.6 × 10^−3^	60	0.3	80	41.7
**Sputtering**	Cu	Ar	≤1 × 10^−5^	3.6 × 10^−3^	60	0.3	80	25.0

**Table 2:** Layer sequences and thicknesses of the Cu/Sn multilayer configurations.

Configuration	Layer Sequence	Total Sn Thickness (μm)	Total Cu Thickness (μm)	Total Interlayer Thickness (μm)
**3layer**	Sn/Cu/Sn	5.0	1.5	6.5
**3layer/3layer**	(Sn/Cu/Sn) × 2	10.0	3.0	13.0
**5layer/5layer**	(Sn/Cu/Sn/Cu/Sn) × 2	15.0	6.0	21.0
**7layer/7layer**	(Sn/Cu/Sn/Cu/Sn/Cu/Sn) × 2	20.0	9.0	29.0

## Data Availability

The original contributions presented in this study are included in the article. Further inquiries can be directed to the corresponding author.
